# Recent advances on Hippo-YAP pathway in skin diseases

**DOI:** 10.3389/fimmu.2025.1728064

**Published:** 2026-01-02

**Authors:** Xuxia Cai, Kexin Chen, Xiahong Li, Kaoyuan Zhang, Jiaxu Gu, Changbing Shen, Bo Yu, Cong Huang

**Affiliations:** 1 Department of Dermatology, Peking University Shenzhen Hospital, Shenzhen, China; 2 Shenzhen Key Laboratory for Translational Medicine of Dermatology, Shenzhen Peking University - The Hong Kong University of Science and Technology Medical Center, Shenzhen, China; 3 PKU-Shenzhen Clinical Institute of Shantou University Medical College, Shantou University, Shantou, China

**Keywords:** Hippo-YAP pathway, inflammatory skin diseases, skin aging, skin tumors, wound healing

## Abstract

The Hippo-YAP pathway is a critical signaling cascade that regulates essential cellular processes, including proliferation, inflammation, and the fate of cancer cells. Recent studies have increasingly highlighted the significant role of the Hippo-YAP pathway in the pathogenesis and progression of various skin diseases. This review aims to summarize the latest advancements related to the Hippo-YAP pathway in skin disorders, exploring its regulatory mechanisms in the context of skin tumors, inflammatory skin diseases, skin aging, wound healing and skin development. Furthermore, this review will analyze the current challenges and knowledge gaps within this field and propose future directions and potential therapeutics targeting the Hippo-YAP pathway to improve treatment outcomes for skin diseases. This review also acknowledges several limitations, including the heterogeneity of existing studies, variations in experimental models, and the scarcity of clinical evidence directly linking Hippo-YAP dysregulation to specific skin diseases. These factors highlight the need for more standardized and clinically oriented research in the future.

## 1 Introduction

The Hippo-YAP signaling pathway is a critical regulatory network that governs cellular growth, apoptosis, and tissue homeostasis (1–3). This pathway, which includes key components such as the Hippo kinases and Yes-associated protein (YAP), has garnered significant attention in recent years due to its involvement in skin diseases. The dysregulation of this pathway has been linked to a plethora of skin disorders, including skin tumors, inflammatory skin diseases, and conditions associated with aging and wound healing (4–8). Moreover, understanding the nuances of this pathway is essential for elucidating its role in skin physiology and pathology, thereby providing a foundation for potential therapeutic interventions. Recent studies have revealed the pivotal role of the Hippo-YAP pathway in the pathogenesis of psoriasis. For instance, the upregulation of YAP in psoriatic lesions has been associated with keratinocyte proliferation and inflammation, highlighting its potential as a therapeutic target (4, 5). Furthermore, in melanoma, the interplay between YAP and cuproptosis-related genes has been shown to correlate with tumor prognosis (9–11). In addition to its role in tumorigenesis, the Hippo-YAP pathway is also increasingly recognized for its involvement in skin aging and wound healing (12, 13). The decline in YAP activity with age has been shown to impact epidermal and dermal homeostasis, suggesting that enhancing YAP function may mitigate age-related skin conditions (14–16). Moreover, studies have demonstrated that medical interventions, such as low-temperature argon plasma, can modulate YAP activity to promote skin repair and regeneration, emphasizing the potential therapeutic applications of manipulating this pathway in clinical settings (17). These findings highlight the roles of the Hippo-YAP pathway in maintaining skin integrity and contributing to the pathophysiology of skin diseases, further solidifying its status as a critical target for therapeutic exploration (**Table 1**).

**Table 1:** Key molecular regulators of the Hippo–YAP pathway in diverse skin conditions and their mechanistic roles.

Skin condition	Molecular players mentioned	Mechanistic role	Experimental model	Reference
Melanoma	Fascin	Inhibits MST2; activates TAZ; promotes tumorigenesis and stemness	WM793 melanoma cells	(58, 59)
	RASSF1A	Induces MST2 activation by dissociating MST2 from RAF-1; enhances MST2-LATS1 interaction; promotes apoptosis	Hela cell; MCF7 cells (RASSF1A-deficient)	(60)
	SMAC	Interacting with LATS1 to promote apoptosis	A375 melanoma cells	(63)
	BRAF	Early activation of the Hippo pathway suppresses tumorigenesis, whereas late-stage downregulation of MST2 leads to drug resistance.	SV-40 immortalized melanocytes (Mel-ST cells)	(64)
Basal Cell Carcinoma (BCC)	CCN1/CYR61	YAP downstream target; promotes keratinocyte proliferation	Human epidermal keratinocytes	(37)
	CCN2/CTGF	YAP downstream target; mediates ECM remodeling	Human epidermal keratinocytes	(37)
	JNK-JUN	YAP amplifies pre-existing JNK-JUN signaling to drive tumor progression	ASZ cells	(73)
Cutaneous Squamous Cell Carcinoma (cSCC)	EGFR	YAP activating the RAS pathway through upregulating EGFR	Mice	(45, 46)
	ZEB1	Cooperates with YAP to enhance EMT and drive tumorigenesis	Mice	(83)
	S100A8/A9	Functions with YAP to provide compensatory regulation	HCC94 and FaDu cells	(85)
	WBP2	Drives TEAD activity to promote tumor progression	NHK cells	(87)
Psoriasis	YAP and TEAD4	Upregulated in psoriatic patients and mouse models	IMQ-induced mice; Psoriasis patients	(4, 90, 91)
Atopic Dermatitis	IL-4 and IL-13	Cooperates with YAP to exacerbate keratinocyte activation and inflammation	Mice	(97)
Rosacea	YAP	Upregulated in rosacea	Rosacea patients and mice	(100)
Skin Aging	CCN1/CYR61	YAP downstream target, regulates epidermal renewal	NHK cells	(12)
	CCN2/CTGF	As a YAP target, downregulated in aging skin	NHK cells	(12)
	cGAS-STING pathway	YAP/TAZ reduce senescence by suppressing cGAS-STING activation	Fibroblasts and mice	(111)
	BMAL1	BMAL1-YAP complex activates the NF-κB pathway	Mice	(113)
Wound healing	YAP	Reduced YAP activity impairs wound healing	Mice	(13)

BRAF: v-Raf murine sarcoma viral oncogene homolog B1; BMAL1, Brain and muscle; CCN1/CYR61: Cysteine-rich protein 61; CTGF/CCN2, : Connective tissue growth factor; EGFR: Epidermal growth factor receptor; LATS1: Large tumor suppressor kinase 1; NHK cells: Normal human keratinocytes; RAF-1: RAF proto-oncogene serine/threonine-protein kinase; SMAC: Second mitochondria-derived activator of caspases; TAZ: transcriptional coactivator with PDZ-binding motif; TEAD: Transcriptional enhanced associate domains; WBP2: WW-binding protein 2; YAP: Yes-associated protein; ZEB1: Zinc finger E-box-binding homeobox 1.

This review aims to systematically explore the current knowledge surrounding the Hippo-YAP pathway in the context of skin disorders, including its implications for skin tumor biology, inflammatory conditions, aging processes, wound healing mechanisms, and skin development. By collating and analyzing recent findings, this review seeks to provide a valuable resource for researchers and clinicians alike, ultimately contributing to the advancement of targeted therapies that leverage the Hippo-YAP pathway for improved skin health outcomes.

## 2 Basic concepts for Hippo-YAP pathway

### 2.1 Hippo-YAP pathway: components and functions

**Figure 1:**
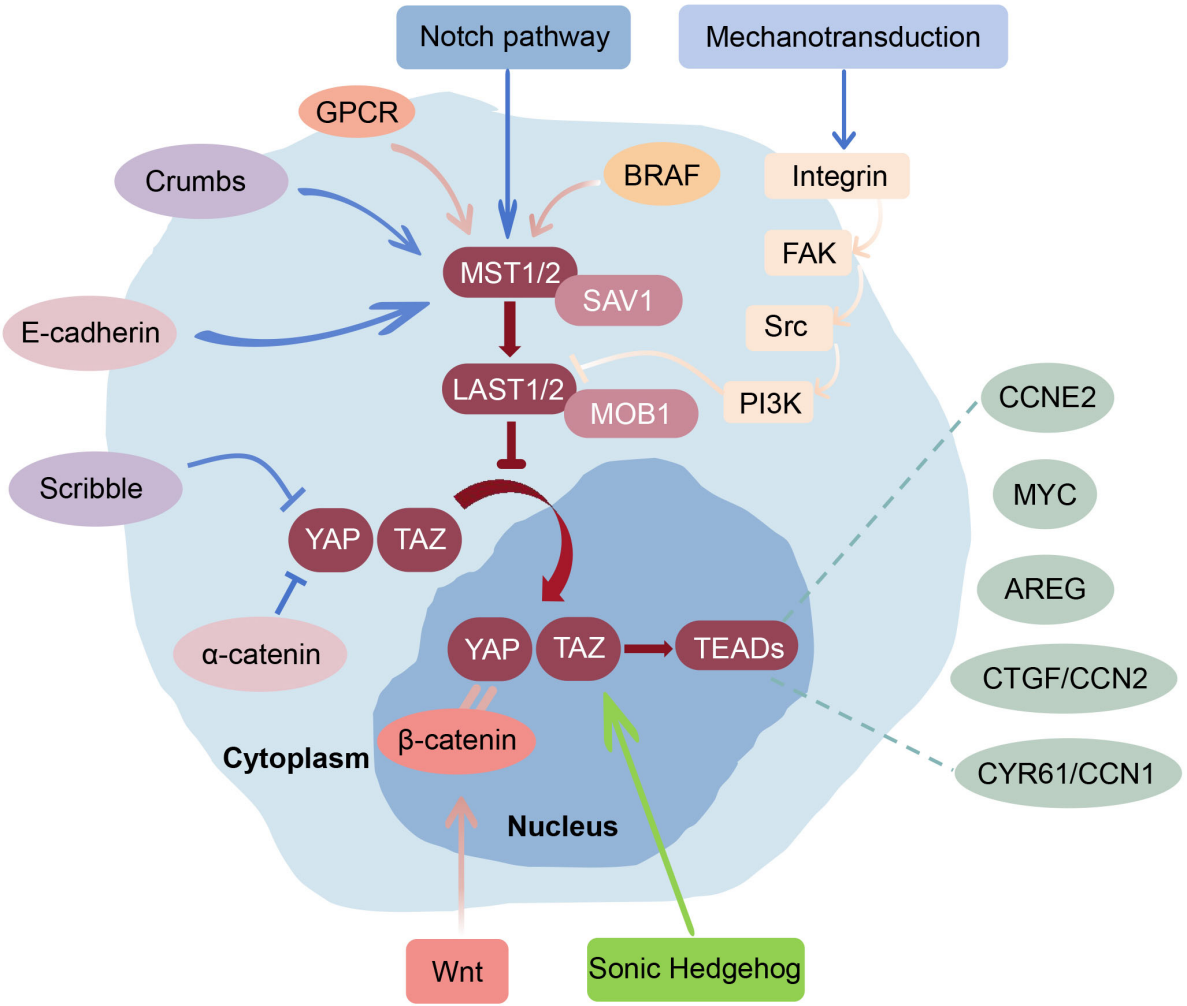
Key regulatory mechanisms and signaling integration of the Hippo-YAP pathway in the skin. Cell-cell junction proteins (E-cadherin, α-catenin) and polarity complexes (Scribble, Crumbs) regulate the Hippo-YAP pathway by modulating cell adhesion and cytoskeletal tension. Meanwhile, SAV1 and MOB1 could interact with MST1/2 and LATS1/2, respectively, to activate this kinase cascade. When Hippo kinases are inhibited, YAP/TAZ translocate into the nucleus and bind TEADs to induce target genes involved in cell proliferation (e.g., CCNE2, MYC), paracrine signaling (e.g., AREG), and ECM remodeling (e.g., CTGF/CCN2, CYR61/CCN1). Mechanical cues could regulate YAP/TAZ through the Integrin-FAK-Src-RhoA axis and PI3K/Akt signaling. The Hippo-YAP pathway also integrates input from Wnt/β-catenin, RAS-MAPK, Sonic Hedgehog, PI3K/Akt, Notch, and GPCR pathways, collectively coordinating proliferation, differentiation, ECM remodeling, and immune responses in skin homeostasis and disease.

The Hippo-YAP signaling pathway is a highly conserved network that plays a critical role in controlling cell proliferation and apoptosis, thereby maintaining tissue homeostasis (18, 19). At its core, the Hippo pathway consists of a cascade of kinases, primarily the Hippo kinases (MST1/2) and the large tumor suppressor kinases (LATS1/2), which are activated in response to various intracellular and extracellular signals, including cellular energy status, cell density, hormonal signals, and mechanical stimuli (1). MST1/2 interact with the scaffold protein SAV1 to phosphorylate and activate LATS1/2, while MOB1 serves as an essential co-activator. Activated LATS1/2 subsequently phosphorylate YAP and PDZ-binding motif (TAZ), preventing their nuclear accumulation and suppressing the expression of proliferation-associated genes (20). The intracellular location of YAP/TAZ determines its transcriptional activity. Phosphorylated YAP/TAZ are retained in the cytoplasm through their interaction with 14-3–3 proteins, after which they undergo proteasomal degradation mediated by β-transducin repeat-containing protein (β-TrCP) (21, 22). However, when the Hippo pathway is inhibited, YAP accumulates in the nucleus, where it binds to transcription factors such as transcriptional enhanced associate domains (TEADs) to activate the transcription of target genes that promote cell growth and inhibit apoptosis (20) (**Figure 1**). This regulatory mechanism is crucial for maintaining skin homeostasis, as YAP influences the behavior of keratinocytes and fibroblasts, which are essential for skin integrity and repair (12, 14, 23, 24). In addition, as a tumor suppressor pathway, activation of the Hippo cascade leads to the inactivation of YAP/TAZ, thereby preventing excessive cell proliferation and maintaining proper organ size; conversely, aberrantly activated YAP/TAZ can promote tumor development (25–28).

### 2.2 Regulatory mechanisms of Hippo-YAP pathway

Beyond the core kinase cascade, the regulation of the Hippo-YAP pathway also involves complex interactions between upstream signals, core signaling components, and downstream transcriptional mechanisms (**Figure 1**). For example, cell-cell junction proteins (such as E-cadherin and α-catenin) and cell polarity complexes (including the Scribble and Crumbs complexes) regulate the Hippo-YAP pathway by modulating cell adhesion and cytoskeletal tension (29–33). Specifically, silencing E-cadherin markedly reduces MST1/2 and LATS1/2 expression while significantly increasing YAP expression (33); deletion of α-catenin in mouse cardiomyocytes enhances nuclear YAP localization (29); Scribble suppresses YAP-induced pronephric cyst formation in zebrafish (30); and Crumbs functions as a tumor suppressor that activates the Hippo pathway by binding to Expanded (31). Downstream, YAP/TAZ function primarily through binding to TEAD transcription factors, inducing a wide range of target genes involved in cell proliferation (e.g., CCNE2, MYC), extracellular matrix remodeling (e.g., CTGF/CCN2, CYR61/CCN1), and paracrine signaling (e.g., AREG), thereby exerting multifaceted roles in skin regeneration and tumorigenesis (34–37).

In addition to canonical kinase cascades, the transcriptional activity of YAP/TAZ is profoundly regulated by mechanotransduction. Cells sense and respond to changes in extracellular mechanical cues, such as extracellular matrix (ECM) stiffness, cell geometry, and intercellular tension, through cytoskeletal remodeling and Rho/ROCK-mediated actomyosin contractility (38). Under conditions of high cell density, strong intercellular adhesion, or a soft ECM, the Hippo kinase cascade is activated, resulting in the cytoplasmic retention and degradation of YAP/TAZ. Conversely, low cell density or culture on a stiff ECM suppresses Hippo kinase activity, thereby promoting the nuclear translocation of YAP/TAZ (39). Notably, the regulation of YAP activity by mechanotransduction is primarily mediated through the Integrin-FAK-Src-RhoA signaling axis, with the PI3K pathway potentially contributing as an auxiliary modulator (14). A stiff ECM promotes actin polymerization and stress fiber formation via activation of Integrin-FAK signaling, thereby suppressing the Hippo kinase cascade and driving YAP/TAZ nuclear translocation (16). Consistently, pharmacological inhibition of FAK or Src under low cell density conditions prevents growth factor-induced YAP nuclear localization, further supporting the essential role of FAK/Src signaling in the mechanical regulation of YAP activity (40).

### 2.3 Integration of Hippo-YAP signaling with other pathways

The Hippo-YAP pathway interacts with multiple critical signaling cascades to regulate cellular proliferation, differentiation, and tissue homeostasis. Among these, the Wnt/β-catenin pathway is one of the most extensively characterized partners and has been implicated in the pathogenesis of several skin diseases, including melanoma and basal cell carcinoma (41–44). The RAS cascade also functionally intersects with Hippo-YAP signaling, contributing to melanoma development and potentially influencing other skin disorders (45, 46). In addition, the Sonic Hedgehog (Shh) pathway has been shown to act cooperatively with YAP/TAZ to promote skin tumorigenesis (47). Beyond these major pathways, additional signaling networks—such as PI3K/Akt, Notch, and G protein-coupled receptor (GPCR) pathways—can modulate Hippo-YAP activity in specific developmental or pathological contexts (43, 46, 48, 49). A more detailed discussion of these interactions is provided in the subsequent sections.

As the largest organ of the human body, the skin is susceptible to a variety of diseases, including tumors, inflammatory disorders, aging-related conditions, wound-healing defects, and appendage developmental abnormalities. Increasing evidence indicates that the Hippo-YAP pathway plays a critical role in the initiation and progression of these conditions by regulating cellular proliferation, differentiation, regeneration, and the local immune environment (**Figure 1**). Therefore, elucidating the functions of the Hippo-YAP pathway in both physiological and pathological skin states is essential for advancing mechanistic understanding and developing new therapeutic strategies for skin diseases.

## 3 Hippo-YAP pathway and skin tumors

### 3.1 Hippo-YAP pathway in melanoma

The Hippo-YAP pathway is increasingly recognized as a critical regulator in the pathogenesis of melanoma, the most aggressive form of skin cancers. Notably, the overall expression of YAP is frequently elevated in melanoma tissues, and this upregulation is associated with poor prognosis and increased tumor aggressiveness (50–53). Additionally, nuclear localization of YAP has been identified as an independent risk factor for distant metastasis in melanoma (54). Further, the loss of upstream regulators of the Hippo-YAP pathway, such as SAV1 and LATS1, leads to uncontrolled YAP activation and cancer cell growth in melanoma (55). Epithelial-mesenchymal transition (EMT) is a fundamental biological program, characterized by loss of epithelial features, reduced cell–cell adhesion, and acquisition of mesenchymal traits (56). Accumulating evidence indicates that melanoma progression involves an EMT-like phenotypic switch, in which tumor cells exhibit decreased adhesion, enhanced motility, and increased invasive capacity (56). Interestingly, YAP overexpression has been shown to promote this EMT-like transition, thereby increasing melanoma cell proliferation and metastatic potential (57).

**Figure 2:**
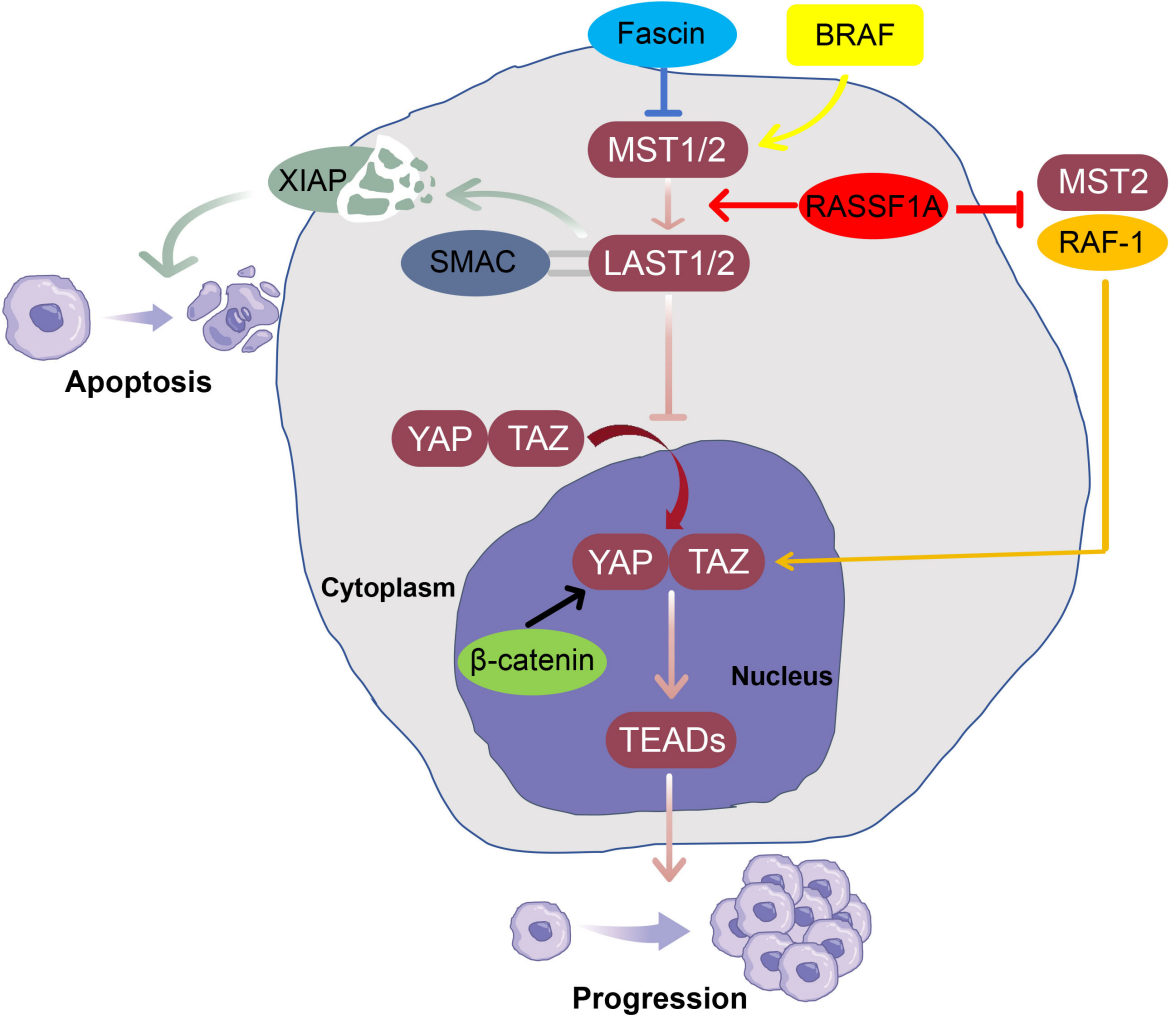
Key regulatory mechanisms of the Hippo-YAP pathway in melanoma. The activation of the MST-LATS kinase cascade (Hippo kinases, MST1/2 and LATS1/2) inhibits the activity of YAP/TAZ and prevents their nuclear translocation. When Hippo kinases are inhibited, YAP/TAZ can bind to TEADs, inducing the expression of downstream target genes and promoting melanoma progression. Mechanistically, Fascin suppresses MST2 activity, thereby reducing LATS1/2 activation and enhancing YAP-driven melanoma progression. In contrast, RASSF1A promotes apoptosis by inhibiting the RAF-1-MST2 interaction and enhancing MST2-LATS1 signaling. SMAC interacts with LATS1 to facilitate XIAP degradation, relieving XIAP-mediated inhibition of apoptosis. Additionally, oncogenic BRAF induces early growth arrest in melanocytes by activating the Hippo kinases and promoting YAP phosphorylation. Together, these mechanisms illustrate the major upstream regulators and downstream outcomes of Hippo-YAP dysregulation in melanoma.

Several molecular regulators have been identified as possible modulators of Hippo-YAP pathway in the pathogenesis of melanoma (**Figure 2**). Fascin, an actin-bundling protein, is upregulated in melanoma and contributes to tumorigenesis and the maintenance of cancer cell stemness by inhibiting the Hippo kinase MST2 and promoting the activation of the transcriptional co-activator TAZ in WM793 melanoma cells (58, 59). RASSF1A is a tumor suppressor in melanoma. *In vitro*, it inhibits the interaction between the RAF proto-oncogene serine/threonine-protein kinase (RAF-1) and MST2 while enhancing the interaction between MST2 and LATS1, thereby activating the pro-apoptotic signaling (60). Interestingly, although RAF-1 knockdown does not alter MST2 expression levels, it significantly reduces the expression of YAP and TAZ, thereby suppressing cell proliferation, migration, and invasion, while promoting apoptosis in four melanoma cell lines (61). X-linked inhibitor of apoptosis protein (XIAP), a member of the inhibitor of apoptosis (IAP) family, is upregulated in melanoma cells and is associated with resistance to chemotherapy and radiotherapy (62). In A375 melanoma cells, the second mitochondria-derived activator of caspases (SMAC) promotes apoptosis by interacting with LATS1, leading to the degradation of X-linked inhibitor of apoptosis protein (XIAP) in metastatic malignant melanoma (63).

The RAS-RAF-MEK-ERK signaling cascade is hyperactivated in the majority of malignant melanomas. In the early stages of melanomagenesis, oncogenic v-Raf murine sarcoma viral oncogene homolog B1 (BRAF) has been shown to promote the growth arrest and benign nevus formation of immortalized melanocytes (Mel-ST cells) by activating the Hippo-YAP pathway (64). However, during melanoma progression and therapeutic resistance, Hippo-YAP signaling becomes disrupted. In BRAF inhibitor-resistant melanoma cells, the Hippo component MST2 is downregulated (65), while nuclear localization of YAP is increased (66), leading to drug-resistance. This dual role highlights the context-dependent regulation of the Hippo pathway at different stages of melanoma development.

Furthermore, YAP interacts with other oncogenic signaling networks to promote the progression of melanoma. Notably, the interaction between YAP and the Wnt/β-catenin pathway has been shown to exacerbate tumor progression. β-catenin is an interaction partner of YAP on DNA in melanoma cells, and the β-catenin-YAP complex alters transcriptional programs, shifting them from senescence-stabilizing gene expression toward a tumor-supportive profile (67).

Together, these findings underscore the complexity of the Hippo-YAP pathway governing melanoma initiation, progression, and resistance to therapy. Further, the understanding of its role in melanoma has opened avenues for targeted therapeutics, thereby providing a novel approach to melanoma management (11).

### 3.2 Hippo-YAP Pathway in non-melanoma skin cancers

Cutaneous non-melanoma skin cancers (NMSCs) are primarily composed of basal cell carcinoma (BCC) and cutaneous squamous cell carcinoma (cSCC), which represent the two most common keratinocyte-derived malignancies (68). Studies have revealed that Hippo-YAP signaling is aberrantly regulated in BCC and cSCC, driving tumor progression and enhancing invasion behavior (69, 70).

Genomic analysis of BCC has revealed recurrent mutations in Hippo regulators and upregulation of YAP target genes (71). Meanwhile, YAP is markedly upregulated in BCC, and tumor cells display both strong cytoplasmic and nuclear YAP localization, indicating aberrant activation of the YAP expression. Additionally, both upregulated YAP expression and enhanced nuclear accumulation have been associated with increased tumor cell invasiveness (37, 72). Notably, the conditional deletion of both YAP and TAZ has been shown to abrogate tumor formation in transgenic mouse models (73, 74), confirming their role in BCC progression. The CCN protein family, particularly cysteine-rich protein 61 (CYR61/CCN1) and connective tissue growth factor (CTGF/CCN2), have been identified as downstream transcriptional targets of YAP and are markedly upregulated in keratinocytes within BCC tumor nests (37). Functionally, CYR61/CCN1 promotes keratinocyte proliferation and survival, while CTGF/CCN2 contributes to stromal activation and ECM remodeling by stimulating collagen synthesis (12, 75). Consequently, YAP knockdown reduces CCN1/CCN2 expression, suppresses keratinocyte growth, and potentially inhibits stromal remodeling in BCC (37).

Given that YAP cooperates with multiple developmental pathways during skin tumorigenesis, considerable attention has been directed at its interaction with Wnt/β-catenin signaling. Dysregulation of the Wnt/β-catenin pathway has been associated with BCC pathogenesis as well as skin growth, development, and repair (76, 77). Nuclear localization of β-catenin is a characteristic feature of basal cell carcinoma, and basal cell carcinomas with nuclear β-catenin positivity exhibit markedly increased proliferative capacity, suggesting that nuclear β-catenin contributes to the pathogenesis of BCC (78). Under Wnt-inactive conditions, YAP and TAZ are incorporated into the β-catenin destruction complex with Axin, promoting the degradation of β-catenin. In this state, YAP/TAZ remain in the cytoplasm and function as negative regulators of Wnt signaling. Conversely, activation of the Wnt receptor—such as through LRP6—drives Axin recruitment to the receptor complex, releasing YAP/TAZ from the destruction machinery. As a result, β-catenin becomes stabilized and accumulates in the nucleus, while YAP/TAZ are released from the complex and become available to participate in Wnt-induced transcriptional activation (79).

In addition, activation of the Sonic Hedgehog (Shh) signaling pathway is a major pathogenic event driving the development of BCC (77). In a mouse model of hedgehog-smoothened-driven BCC, inhibition of TEAD activity or knockdown of YAP/TAZ leads to rapid activation of differentiation programs and the elimination of tumor cells. This demonstrates that YAP/TAZ-TEAD signaling is essential for suppressing differentiation and maintaining BCC proliferation driven by oncogenic Hedgehog signaling (47).

Despite the well-established cooperation between the YAP/TAZ, Wnt/β-catenin, and Shh pathways (80), recent genetic studies have demonstrated that YAP-driven tumor growth in BCC can occur independently of canonical Wnt and Hedgehog signalings (73). The Hippo-YAP pathway acts as an independent initiator of BCC tumorigenesis in a mouse BCC cell line (ASZ), wherein YAP amplifies a pre-existing JNK-JUN signaling network to promote tumor progression (73). Pre-clinically, inhibition of BCC cell proliferation by the JNK inhibitor SP600125 indicates that targeting the JNK-JUN pathway may represent an effective strategy to interfere with YAP-driven tumorigenesis (81).

YAP also acts as a key driver in the development of cSCC and its aggressive variant, spindle cell carcinoma (spSCC) (82, 83). Studies have confirmed that YAP expression is elevated in cSCC samples across different stages compared to normal skin. Immunohistochemical analyses have shown both cytoplasmic and nuclear YAP staining in cSCC, with several reports indicating predominantly nuclear localization in invasive lesions, which is closely associated with disease progression (82, 83). Overexpression of YAP in mice activates the RAS pathway by upregulating the transcription of epidermal growth factor receptor (EGFR) ligands, such as EGF and amphiregulin (AREG) (45, 46). In contrast, knockdown of YAP in cSCC cell lines (A431 and SCL-1) inhibits cell proliferation, promotes apoptosis, and reduces the invasive and migratory abilities of cSCC cells (82). Additionally, YAP overexpression under two apoptosis-inducing conditions—suspension culture and high cell density—has been shown to promote apoptosis in cSCC cells (84, 85). Moreover, YAP cooperates with zinc finger E-box-binding homeobox 1 (ZEB1), a key transcription factor of EMT, to drive the formation of spSCC in mice by promoting the EMT process (83). Recent studies have demonstrated that S100A8 and S100A9, two heterodimeric members of the S100 family, are implicated in inflammation and tumor progression (86). Notably, activation of the Hippo-YAP pathway induces the co-expression and co-localization of S100A8/S100A9 in SCC cells (85). Mechanistically, S100A8/S100A9 and YAP exert compensatory regulatory functions under different cellular microenvironments, both acting as positive regulators of cell proliferation and negative regulators of cell differentiation (85).

Genome-wide screening has identified YAP and its cofactor WBP2 as cooperative regulators that promote clonal expansion of cSCC stem cells by driving TEAD-mediated transcription of proliferation-related genes (87). As discussed above, mechanotransduction plays a critical role in regulating YAP transcriptional activity. The nuclear exclusion of YAP and WBP2 observed in the densely packed central region of mature normal human keratinocyte (NHK) colonies suggests that contact inhibition contributes to the regulation of downstream proliferation-related genes. Similarly, defective intercellular adhesion in cSCC weakens the inhibitory control over YAP, thereby promoting uncontrolled tumor cell proliferation (87).

These findings suggest that Hippo-YAP pathway and its associated signalings are crucial regulators in the pathogenesis of NMSCs. Importantly, the development of small molecules or biologics capable of inhibiting YAP or activating Hippo activity offers a promising direction for intervening in various skin malignancies, with the potential to improve patient outcomes and survival rates.

## 4 Role of Hippo-YAP pathway in inflammatory skin diseases

### 4.1 Role of Hippo-YAP pathway in psoriasis

**Figure 3:**
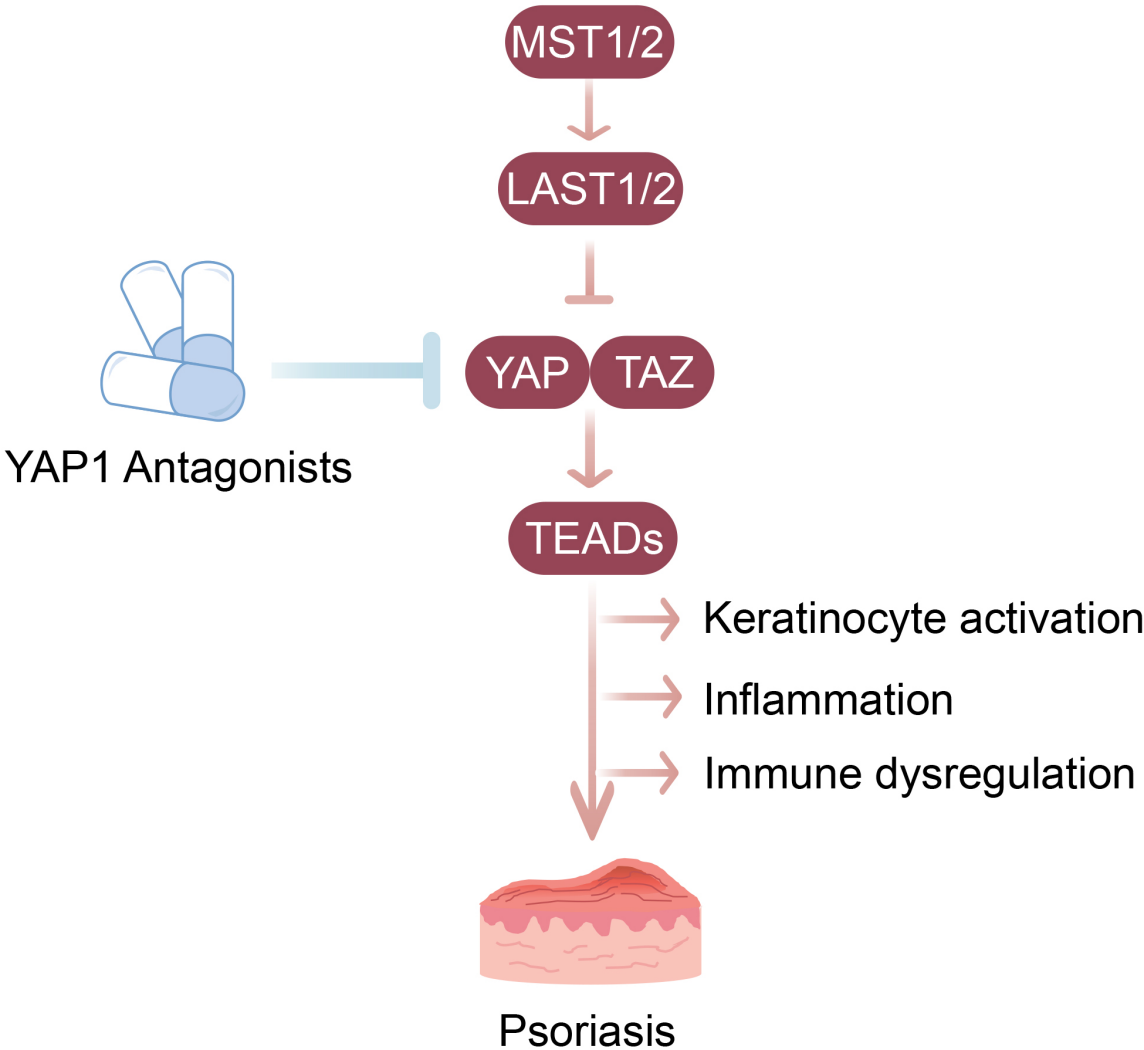
Dysregulated Hippo-YAP signaling contributes to keratinocyte hyperproliferation and inflammation in psoriasis. In psoriatic lesions, Hippo kinases (MST1/2 and LATS1/2) are downregulated, while YAP/TAZ-TEADs transcriptional complexes are upregulated. Activated YAP/TAZ-TEADs axi promotes keratinocyte proliferation and induces inflammatory mediators, contributing to immune dysregulation and disease progression. Therefore, YAP1 antagonists, such as verteporfin (VP), suppress keratinocyte activation and cytokine production, representing a potential therapeutic target for psoriasis.

Psoriasis is a chronic inflammatory skin condition characterized by excessive proliferation of keratinocytes and aberrant immune responses (88, 89). The exact pathogenesis and etiology of this disease remain unclear. Recent studies have highlighted the significant involvement of the Hippo-YAP pathway in the pathogenesis of psoriasis (4, 90, 91) (**Figure 3**). Specifically, analysis of lesional and non-lesional skin biopsies from patients with psoriasis revealed that TEAD4, a key transcription factor of YAP, is highly expressed in psoriatic lesions (90, 92). In keratinocytes, TEAD4 knockdown reduced the expression of CXCL1, CXCL5, and CXCL8, critical inflammatory mediators involved in the pathogenesis of psoriasis (90, 92). More importantly, YAP is upregulated in psoriatic lesions and a mouse model induced by imiquimod (IMQ), suggesting its potential role in disease progression (4, 93, 94). Functional analyses have demonstrated that YAP promotes the proliferation and inflammatory response of keratinocytes, both of which are typical features of psoriasis (4). Additionally, the application of selective YAP antagonists, such as verteporfin (VP), have shown promise in inhibiting keratinocyte proliferation and the production of inflammatory cytokines *in vitro* and *in vivo*, further supporting the role of this pathway in disease progression (4). These findings underscore the potential of Hippo-YAP pathway as a therapeutic target for psoriasis, providing new avenues for managing this debilitating condition.

### 4.2 Role of Hippo-YAP pathway in atopic dermatitis

Atopic dermatitis (AD) is another prevalent inflammatory skin disease, characterized by skin barrier dysfunction and immune dysregulation, with itching as the main symptom (95, 96). The role of Hippo-YAP pathway in AD has attracted attention, particularly due to its involvement in keratinocyte biology and inflammatory responses (97). Although studies directly linking YAP expression to AD are still limited, there is evidence suggesting that Hippo-YAP pathway may play a role in the pathophysiology of this disease. In AD models, alterations in the Hippo-YAP pathway have been observed (97–99). Additionally, the YAP-specific inhibitor VP can alleviate the severity of symptoms in AD mouse models by blocking inflammatory factors and the JAK-STAT pathway, indicating that YAP contributes to the inflammatory characteristics of the disease (98, 99). Moreover, the regulatory effect of YAP on keratinocyte proliferation and differentiation could impact the integrity of the skin barrier, which is often compromised in AD (98, 99). Furthermore, the interplay between YAP and inflammatory cytokines, such as IL-4 and IL-13, which are elevated in AD, may exacerbate keratinocyte activation and inflammation in mice (97). Therefore, understanding the precise mechanisms by which the Hippo-YAP pathway regulates the pathogenesis of AD could open avenues for targeted therapies to restore skin barrier function and mitigate inflammation.

### 4.3 Role of Hippo-YAP pathway in other inflammatory skin diseases

Apart from psoriasis and atopic dermatitis, the Hippo-YAP pathway is also associated with a variety of other inflammatory skin diseases, such as rosacea. Recent studies have shown that dysregulation of Hippo-YAP signaling may lead to the inflammatory processes observed in this condition. For instance, elevated YAP levels were observed in skin samples from patients with rosacea, compared to healthy donors, suggesting that YAP may play a role in the pathogenesis of rosacea (100). Additionally, in a LL-37-induced rosacea mouse model, treatment with VP alleviated the clinical symptoms of rosacea, highlighting YAP as a potential therapeutic target for this inflammatory skin disease (100).

## 5 Hippo-YAP pathway in skin aging

### 5.1 Overview of skin aging biology

Skin aging is a multifaceted biological process characterized by intrinsic and extrinsic factors leading to structural and functional decline in skin tissues (101). Intrinsically, aging involves genetically programmed factors such as telomere shortening, mitochondrial dysfunction, and cellular senescence marked by increased expression of cyclin-dependent kinase inhibitors. These changes lead to reduced proliferative capacity in epidermal keratinocytes and dermal fibroblasts, contributing to epidermal thinning and dermal atrophy. Extrinsic factors, particularly UV radiation, pollution, and lifestyle choices, exacerbate skin aging by inducing oxidative stress, DNA damage, and chronic inflammation (102, 103). A positive feedback loop exists between chronic skin inflammation and aging. Inflammatory responses amplify the senescence-associated secretory phenotype (SASP), reshaping the skin microenvironment and thereby accelerating the aging process. In turn, aging promotes the secretion of proinflammatory cytokines, leading to growth arrest and the establishment of inflammatory senescence (104). Furthermore, aberrant activation of the cGAS-STING pathway is a common feature of senescent cells and contributes to SASP amplification (105). Recent findings show that imbalanced nucleotide metabolism can trigger mitochondrial DNA release and activate cGAS-STING–dependent inflammatory signaling in senescent cells, further driveing SASP production and reinforces the positive feedback loop of chronic inflammation and aging (106). Understanding these biological mechanisms is crucial for developing effective anti-aging strategies and interventions aimed at preserving skin health and functionality as individuals age.

### 5.2 Regulation of skin aging by the Hippo-YAP pathway

Recent studies have highlighted the central role of the Hippo-YAP pathway in regulating key cellular processes associated with skin aging. The Hippo-YAP pathway maintains homeostasis within multicellular organisms and regulates skin collective aging by controlling the balance between keratinocyte proliferation and apoptosis (107). During aging, reduced YAP/TAZ signaling diminishes proliferative capacity and increases cellular senescence, leading to epidermal thinning and disrupted skin cell turnover, thereby accelerating tissue aging (13). This process is likely mediated, at least in part, by the YAP target gene CYR61/CCN1. Beyond the regulation of cell proliferation and survival, the Hippo-YAP axis also contributes to skin aging by maintaining ECM homeostasis through its downstream targets. In human dermal fibroblasts, the expression of YAP/TAZ and their downstream effector, CTGF/CCN2, decreases with age (12). This downregulation of CTGF/CCN2 is regulated by YAP/TAZ (108). Given the critical role of CTGF/CCN2 in maintaining ECM integrity and the fact that its expression in dermal fibroblasts is strongly enhanced by transforming growth factor-β (TGF-β) signaling, recent study indicated that YAP cooperate with the TGF-β–SMAD axis to promote CTGF transcription (109). Meanwhile, in the absence of exogenous TGF-β, YAP/TAZ can induce pro-fibrotic effects in response to matrix stiffness, suggesting that they exert broader regulatory functions during fibrogenesis (12, 75, 110).

In addition to its role in maintaining ECM integrity, YAP/TAZ also exert anti-aging effects by suppressing inflammatory responses (12). Recent studies have demonstrated that YAP and TAZ function as key mechanosensitive regulators that suppress aberrant activation of the cGAS-STING pathway in fibroblasts and YAP/TAZ-deficient mouse models (111, 112). Loss or mechanical inhibition of YAP/TAZ compromises nuclear envelope integrity, leading to leakage of genomic DNA and subsequent recruitment and activation of cGAS at sites of nuclear rupture. This results in STING-dependent induction of inflammatory and senescence-associated genes, whereas genetic depletion of cGAS or STING abolishes the inflammatory program triggered by YAP/TAZ loss. These findings establish that the Hippo-YAP pathway restrains chronic inflammation and aging by suppressing unintended cGAS-STING activation (111). Brain and muscle Arnt-like protein-1 (BMAL1) plays a critical role in maintaining epidermal homeostasis, and its deficiency leads to premature aging. Recent studies have shown that BMAL1 can form a complex with YAP in the aged epidermis, and the BMAL1-YAP complex binds to enhancer elements of inflammation-related genes, thereby promoting the expression of NF-κB-dependent inflammatory target genes (113). Collectively, YAP/TAZ function as key modulators linking mechanotransduction, inflammation, and aging in the skin.

### 5.3 Therapeutic modulation of Hippo-YAP signaling in skin aging

Given the crucial role of the Hippo-YAP pathway in skin aging, several intervention strategies have been explored to enhance YAP/TAZ activity and promote skin rejuvenation. One promising approach involves the use of pharmacological agents that can inhibit the upstream regulators of the Hippo-YAP pathway. For instance, small molecules that inhibit the MST/LATS kinases, which are responsible for phosphorylating and inactivating YAP, have shown potential in preclinical studies (114, 115). Interestingly, exposure of keratinocytes to hydrogen peroxide leads to enhanced nuclear accumulation of YAP, whereas treatment with antioxidants after hydrogen peroxide stimulation suppresses total YAP expression, suggesting that antioxidants may help regulate YAP activity and thereby promote skin renewal (116). Moreover, emerging technologies such as gene therapy and regenerative medicine, including the use of stem cells, offer innovative avenues for enhancing YAP/TAZ signaling in aged skin (117). These findings highlight the therapeutic potential of targeting Hippo-YAP signaling to restore the regenerative capacity of skin.

## 6 Hippo-YAP pathway in wound healing

### 6.1 Physiological process of wound healing

Wound healing is a complex physiological process of tissue reconstruction, involving a series of well-coordinated events, including hemostasis, inflammation, proliferation, and remodeling. During this process, the mechanical stress on the tissue and the immune response generated interact to determine the manner of healing (118). Upon injury, the first response is the hemostatic reaction, which forms a blood clot as a temporary barrier against pathogens. Then, it enters the inflammatory phase, where immune cells are recruited to the injured site to clear debris and pathogens (118). The proliferation phase involves the migration and proliferation of keratinocytes, fibroblasts, and endothelial cells, which are crucial for re-epithelialization, extracellular matrix deposition, and angiogenesis. The final remodeling phase involves the maturation of collagen and other ECM components. The Hippo-YAP pathway plays key roles in regulating these processes, especially in the cell proliferation and migration involved in wound healing (13). Thus, the dysregulation of this pathway can lead to impaired healing or pathological conditions such as fibrosis and chronic wounds (119).

### 6.2 Role of Hippo-YAP in wound healing

Recent studies have emphasized the importance of Hippo-YAP pathway in multiple aspects of cell behavior during wound healing, including cell proliferation, migration, and EMT (120, 121). Normally, YAP exists both in the nucleus and cytoplasm of skin cells. However, in the context of wound healing, YAP is activated in response to mechanical cues and mostly enters the nucleus. Knockdown of YAP and TAZ markedly delayed the rate of wound closure in mice (13). In addition, recent evidence highlights the central role of YAP/TAZ in regulating fibroblast activation and maintaining the balance between regenerative healing and fibrotic scar formation (13, 122). TGF-β1 is a key driver of myofibroblast differentiation and wound contraction (13). Genetic deletion of YAP/TAZ markedly reduces TGF-β1 expression, resulting in impaired fibroblast activation (13). Consistent with this, excessive nuclear accumulation of YAP has been strongly linked to fibrotic scar formation. Under non-fibrotic conditions, cytoplasmic YAP is sequestered through its interaction with α-catenin, preventing its translocation to the nucleus. However, in pro-fibrotic environments, increased caspase-3 activity cleaves α-catenin, leading to the release and nuclear translocation of YAP. Nuclear YAP subsequently drives the transition of fibroblasts from a regenerative phenotype to a pro-fibrotic state, thereby promoting scar formation (122).

Emerging evidence has further highlighted the importance of mechanotransduction-dependent YAP activation in determining fibroblast fate during wound healing. Mechanical tension can activate YAP in dermal fibroblasts to drive the emergence of Engrailed-1–positive profibrotic fibroblasts, thereby promoting scar formation (123). Conversely, disrupting YAP-mediated mechanotransduction redirects wound healing toward a regenerative trajectory, partly through activation of Trps1- and Wnt-related programs (124). Notably, inhibition of YAP using verteporfin has been shown to prevent scarring and promote regeneration in a porcine wound model and human xenografts, underscoring the translational potential of targeting mechanotransduction in wound repair (125).

Moreover, the interaction between Hippo-YAP and other signaling pathways (such as TGF-β and β-catenin) highlights its role in coordinating the complex cellular responses required for wound healing. In diabetic models, wound healing is usually impaired, and the inactivation of YAP is associated with improved healing outcomes (121), indicating that therapeutic strategies targeting YAP may be beneficial for the wound repair of patients with chronic wounds.

### 6.3 Application of Hippo-YAP pathway in wound healing

The therapeutic potential of targeting Hippo-YAP pathway in wound healing is gaining attention, particularly in the context of chronic wounds and diabetic ulcers where healing is often compromised. The use of medical gas plasma and vitamin D3 analogs has been shown to promote wound healing by modulating YAP activity (120, 121). Calcipotriol, a synthetic vitamin D3 derivative, has been shown to enhance keratinocyte migration and induce an EMT-like phenotype through activation of the Hippo-YAP pathway *in vitro*, and topical application accelerated re-epithelialization in a mouse wound model (120). VP, a selective YAP inhibitor, has been reported to exert therapeutic effects in inflammatory skin disorders, while also attenuating fibrosis and promoting scar-free skin regeneration through inhibition of TGFβ-induced actin stress fiber formation in dermal fibroblasts (126). Copper(II)-2-acetylpyridine bis (CuATSM) significantly enhances angiogenesis, cell proliferation, and collagen deposition during cutaneous wound healing in mice, while also reducing scar formation. In parallel, CuATSM markedly suppresses the nuclear localization of YAP, suggesting that its pro-healing effects may be mediated through modulation of the Hippo-YAP pathway (127). Additionally, the integration of YAP-targeting strategies with existing wound care practices could significantly improve healing rates and outcomes for patients suffering from chronic skin conditions. As research continues to elucidate the precise mechanisms by which YAP influences wound healing, the prospect of developing targeted therapies that harness this pathway could revolutionize the management of skin injuries and disorders, ultimately leading to better patient care and improved quality of life.

## 7 Hippo-YAP pathway in skin development

During skin development, YAP/TAZ exhibit a dynamic pattern of subcellular localization. In the early embryonic epidermis, before stratification occurs, YAP/TAZ are broadly expressed and predominantly localized in the nucleus (128). As epidermal differentiation proceeds, YAP becomes differentially distributed and is largely restricted to basal layer cells (14, 128, 129). Functional studies demonstrate that YAP/TAZ serve dual roles in promoting basal cell proliferation while restraining differentiation. YAP activation drives TEAD-dependent transcriptional programs that upregulate cell cycle and growth-related genes, as well as effectors of the EGFR-RAS and integrin pathways, with CTGF and CYR61 validated as direct YAP targets and key mediators of epidermal growth (14, 128, 130). Consistent with these transcriptional effects, enforced YAP expression in mouse embryos induces excessive proliferation, impaired differentiation, and epidermal hyperplasia with defective hair follicles, ultimately leading to perinatal lethality (128). In contrast, epidermal-specific deletion of YAP/TAZ reduces basal cell proliferation in adult mice (14). The Notch signaling pathway induces growth arrest and promotes keratinocyte differentiation (131), whereas YAP actively suppresses differentiation by inhibiting the Notch pathway, thereby maintaining basal cells in an undifferentiated state (128). Recent work has identified the receptor-interacting serine/threonine kinase 4 (RIPK4)-LATS1/2 module as a critical upstream regulatory axis, in which RIPK4 activates LATS1/2 to promote YAP/TAZ phosphorylation and nuclear exclusion, relieving their repression of differentiation-inducing genes. In RIPK4-deficient cells, differentiation markers are markedly reduced, and simultaneous YAP/TAZ knockdown partially restores their expression, confirming YAP/TAZ as central effectors of this pathway (132).

The Hippo-YAP signaling axis also plays a critical role in the development of skin appendages. Immunostaining analyses of adult mouse skin demonstrate that YAP is selectively enriched in the bulge region and interfollicular epidermis (133), suggesting the activation of YAP signaling within the hair follicle and its stem cell niche. Microarray profiling further indicates that nuclear YAP/TAZ activity is tightly associated with the proliferative potential of hair follicles. In proliferative compartments—including the outer root sheath (ORS) and transit-amplifying matrix (Mx) cells at the follicle base—YAP is predominantly localized in the nucleus, whereas in terminally differentiated inner root sheath (IRS) and hair shaft cells, YAP largely shifts to the cytoplasm (128), reflecting its dynamic regulation during follicular proliferation and differentiation. Additionally, the hair follicle bulge contains neural crest-derived stem cells (hfNCSCs) with multipotent differentiation capacity, in which the Hippo-YAP pathway also plays essential functions. Treatment with VP markedly reduces the viability of hfNCSCs, leading to sparse, shrunken cells, indicating that Hippo-YAP signaling is critical for maintaining the proliferative activity of these stem cells (134).

## 8 Roles of Hippo-YAP pathway in cutaneous immunity

Innate immunity constitutes the first line of defense of the skin against external insults; keratinocytes not only provide a structural barrier but also participate in immune responses by sensing pathogens, injury, and inflammatory signals (135). NOD-like receptor family pyrin domain-containing 3 (NLRP3) is an important component of the human innate immune system and plays a central role in mediating various inflammatory skin diseases (136). During cutaneous inflammatory responses, activation of the NLRP3 inflammasome is pivotal, and current studies indicate a close association between YAP and this process. YAP promotes NLRP3 inflammasome activation and enhances the release of inflammatory mediators such as IL-1β (137). These findings suggest that the Hippo-YAP pathway acts as a key mediator in amplifying keratinocyte-driven inflammation, thereby influencing skin innate immune homeostasis.

Beyond keratinocytes, the Hippo-YAP pathway plays crucial roles in multiple innate immune cell types and is closely linked to the initiation and progression of skin inflammation. Numerous studies have shown that under pathological conditions (tumor or inflammatory disease), YAP/TAZ activation drives macrophage recruitment by stimulating the production of various chemokines, such as CCL2 and CXCL1 (138). Moreover, pro-inflammatory stimuli markedly increase YAP and TAZ protein expression in macrophages, indicating a role for YAP/TAZ in macrophage polarization (139). Functional studies reveal that loss of YAP/TAZ impairs pro-inflammatory responses, further supporting their critical role in macrophage-mediated inflammation. In dendritic cells, the core Hippo kinase Mst1 is also closely involved in inflammatory regulation. Mst1 has been shown to suppress IL-6 secretion in dendritic cells, thereby indirectly inhibiting Th17 differentiation (140), implying that the Hippo-YAP pathway influences innate immunity by modulating specific cytokine production.

In adaptive immunity, CD4^+^ and CD8^+^ T cells serve as central effector cells for immune regulation and responses. Studies have shown that YAP is induced during T-cell activation; paradoxically, this induced YAP subsequently inhibits T-cell activation. Further research has demonstrated that YAP suppresses the activation and differentiation of CD4^+^ T cells (141). Collectively, these findings suggest that YAP plays a role in regulating of T-cell responses to maintain adaptive immune homeostasis. Within CD4^+^ helper T-cell subsets, the balance between Th17 and Treg cells is decisive for determining immune outcomes, and YAP/TAZ play important roles in both lineages. YAP/TAZ are upregulated in Th17 cells (142) and promote Th17 differentiation while concurrently suppressing Treg function (141, 143). Thus, the Hippo-YAP pathway integrates multiple regulatory processes in cutaneous immunity and serves as a critical nexus linking skin inflammatory responses with immune homeostasis.

## 9 The application of Hippo-YAP pathway in clinic, future challenges and innovations

### 9.1 The potential use of Hippo-YAP pathway as biomarkers

A growing body of evidence indicates that dysregulation of the Hippo-YAP pathway is closely associated with various skin disorders, including psoriasis, melanoma, and other cutaneous malignancies (4, 50). These findings position components of the Hippo-YAP pathway as attractive candidates for diagnostic and prognostic biomarkers in dermatological practice. Identifying reliable biomarkers can significantly aid in early diagnosis, monitoring of disease progression, and assessment of therapeutic responses.

YAP expression is upregulated in multiple skin cancers, such as melanoma and NMSCs, with increased nuclear localization correlating with enhanced tumor proliferation and metastatic potential. These characteristics suggest that YAP may serve as a biomarker of disease severity and prognosis (51, 66, 72). Immunohistochemical (IHC) analysis and scoring of Hippo components can assist clinicians in stratifying disease severity and devising personalized treatment strategies, ultimately improving patient outcomes (144). In psoriasis, elevated YAP levels are associated with increased keratinocyte proliferation and inflammation, underscoring its potential role as a severity-related biomarker (4). Additionally, differences in YAP staining patterns across subtypes of skin diseases support its utility in accurate diagnosis and treatment stratification (145). Moreover, YAP/TAZ activity influences skin regeneration in a cell-type-dependent manner: reduced YAP/TAZ signaling impairs epithelial repair, while sustained YAP activation in dermal fibroblasts drives fibrotic remodeling, suggesting its potential as an indicator for assessing regenerative capacity in aging or injured skin (13, 122).

The clinical application of Hippo-YAP pathway as biomarkers holds considerable promise, particularly in the era of personalized medicine. As research continues to uncover the regulatory mechanisms of YAP in cutaneous biology, the development of targeted therapies aimed at modulating this pathway becomes increasingly feasible. Despite these promising insights, several challenges remain, including the context-dependent nature of YAP/TAZ activity, a lack of standardized detection protocols, and limited longitudinal clinical data. Nonetheless, the integration of YAP/TAZ assessment into dermatological diagnostics is anticipated to significantly improve disease classification, risk prediction, and personalized therapeutic decision-making in the near future.

### 9.2 Development of drugs related to Hippo-YAP pathway

Recent studies have also highlighted the potential of developing drugs that modulate YAP activity for the treatment of conditions such as psoriasis and skin tumors (**Table 2**). For instance, the selective YAP antagonist VP has shown promise in inhibiting keratinocyte proliferation and inflammatory factor production in IMQ-induced psoriasis models, suggesting that targeting YAP could effectively mitigate the pathogenesis of this disease and prompting clinical trials to evaluate the efficacy of YAP inhibitors in psoriasis patients (4). Meanwhile, the use of YAP inhibitors in patients with skin tumors has also shown potential in reducing tumor volume (146). Additionally, research is ongoing on the combination of YAP inhibitors with other therapeutic agents to enhance the efficacy of existing treatments while reducing side effects (147). Moreover, there are proposals to explore innovative drug delivery systems, such as cold argon plasma, to enhance the therapeutic effect of YAP-targeted drugs and promote wound healing and skin regeneration (17). Overall, these pre-clinical outcomes underscore the therapeutic relevance of the Hippo-YAP pathway in managing skin diseases, paving the way for further investigations into long-term efficacy and safety profiles of these interventions.

**Table 2:** Mechanisms of Hippo-YAP pathway activators and inhibitors and their effects on various skin conditions.

Intervention	Mechanism	Skin condition	Result	Experimental model	Reference
Verteporfin (VP)	YAP antagonist	Psoriasis	Suppresses inflammatory responses	HaCaT cells	(4)
		Rosacea	Alleviates clinical symptoms	Mice	(100)
		Wound healing	Inhibits the formation of actin stress fibers in dermal fibroblasts	Skin fibroblasts Mice	(126)
Calcipotriol	YAP activator	Wound healing	Enhances keratinocyte migration and promotes EMT	HaCaT cells, NHEK cells and mice	(120)
CuATSM	YAP activator	Wound healing	Promotes cutaneous wound repair	Mice	(127)

### 9.3 The model of cooperation with new technologies and multidisciplinary collaboration

The pathogenesis and progression of skin diseases are highly complex, involving a wide array of biological processes such as genetic predisposition, immune dysregulation, inflammation, and tumorigenesis. Therefore, multidisciplinary collaboration among clinicians, basic scientists, pharmacologists, and bioinformaticians is essential for deepening our understanding of how dysregulation of Hippo-YAP pathway contributes to skin pathologies. For example, clinical dermatology provides valuable insights for disease diagnosis and phenotypic characterization of skin disorders potentially linked to alterations in the Hippo pathway, thereby informing laboratory-based investigations (148). In turn, basic medical research further elucidates the molecular mechanisms involved and correlates these findings with clinical presentations, aiding in the identification of potential biomarkers for disease diagnosis (52). In drug development, collaborative efforts between medicinal chemists and molecular biologists have accelerated the discovery of targeted therapies against the Hippo pathway. In recent years, several small molecules—including TEAD inhibitors such as SW-682 and SWTX-143—have been shown to effectively suppress YAP/TAZ-mediated transcriptional programs and exhibit promising antitumor effects in preclinical models (149, 150).

Furthermore, bioinformatics and artificial intelligence (AI) technologies are becoming indispensable tools for studying the Hippo-YAP pathway and its roles in skin diseases. Recent advances in multi-omics and computational biology have provided powerful approaches to dissect the complex regulation of the Hippo-YAP axis in dermatology. Genomic and transcriptomic analyses enable the identification of YAP/TAZ-related biomarkers and downstream gene signatures in various skin disorders (151). Proteomic studies have revealed multiple post-translational modifications (PTMs) of YAP/TAZ, particularly phosphorylation and ubiquitination, which fine-tune their subcellular localization, stability, and transcriptional activity (152). Integrating multi-omics data within a systems biology framework, combined with machine learning and deep learning approaches, allows the construction of YAP-mediated regulatory networks and predictive models to identify key regulatory nodes and potential therapeutic targets. Recent studies have integrated chemical genetic interaction screening and multi-omics analysis with machine learning to quantify Hippo activity and predict YAP/TEAD dependency or pathway interactions (153). In summary, a multidisciplinary framework integrating clinical, molecular, and computational approaches will be pivotal for defining YAP/TAZ function and advancing Hippo pathway-based therapeutic strategies.

### 9.4 Future directions and challenges faced

The Hippo-YAP pathway plays a critical role in the initiation and progression of skin diseases (4, 12), presenting both challenges and opportunities in dermatological research. As the primary effectors of the Hippo-YAP pathway, YAP and TAZ display diverse functions depending on disease contexts and cellular environments, imposing higher demands on conventional research approaches.

Increasing evidence suggests that YAP/TAZ function as double-edged regulators in the skin. For instance, YAP activation promotes wound healing by inducing target genes such as CYR61/CCN1 and CTGF/CCN2, which facilitate keratinocyte migration and fibroblast activation, but it may also contribute to the development of BCC (12, 75). These findings highlight the potential risks associated with long-term modulation of YAP activity: sustained activation may enhance tumorigenic potential, whereas chronic inhibition could impair tissue repair and accelerate skin aging (117). Clarifying the molecular mechanisms that distinguish beneficial from pathological YAP activity will be critical for the safe application of Hippo-targeted therapies in dermatology (154, 155).

Despite these advances, several unresolved limitations continue to constrain the translational potential of Hippo-YAP-targeted interventions. Current pharmacological approaches, including commonly used YAP-TEAD interaction inhibitors, remain associated with photosensitivity and concerns about off-target cytotoxicity (156). Moreover, clinically applicable biomarkers for monitoring YAP activity are lacking. Although quantitative measures of YAP expression or nuclear localization are informative in experimental settings (4), they have not been validated for routine clinical use. Methodological limitations further widen the gap between basic and clinical research, as most findings derive from mouse models that do not fully recapitulate human skin architecture, immune microenvironments, or disease heterogeneity. Finally, clinical trials assessing the long-term safety of interventions that modulate YAP/TAZ signaling—particularly regarding tumorigenic risk—are scarce.

Future research on the Hippo-YAP pathway in skin diseases is poised to explore several key areas, including the elucidation of molecular mechanisms underlying YAP regulation and the identification of novel therapeutic targets. One promising direction involves investigating the interplay between YAP and other signaling pathways, such as the Wnt/β-catenin pathway, which has been shown to interact with YAP in the context of skin tumorigenesis (157). Additionally, the role of YAP in skin aging and its potential as a biomarker for age-related skin conditions warrants further exploration, as recent studies have linked YAP activity to the maintenance of epidermal homeostasis (12, 24). Overall, continued research into the Hippo-YAP pathway is essential for translating basic science discoveries into clinical applications that can improve patient outcomes in skin diseases.

## 10 Conclusion

This review has explored the multifaceted roles of the Hippo-YAP pathway, emphasizing its regulatory impact on cellular processes integral to skin diseases. As we synthesize the current understanding of this pathway, it is essential to consider the diverse research perspectives and findings that contribute to our knowledge base.

The complexity of the Hippo-YAP pathway, with its intricate network of interactions and regulatory mechanisms, presents both challenges and opportunities for research and clinical application. Balancing the various perspectives of research in this field is crucial for advancing our understanding and harnessing the therapeutic potential of this pathway. In light of the growing body of evidence supporting the Hippo-YAP pathway’s involvement in skin disorders, it is imperative to foster interdisciplinary collaboration among researchers, clinicians, and pharmaceutical developers. Such collaborations can facilitate the translation of basic research findings into clinical applications, ultimately leading to innovative treatment strategies for patients suffering from skin diseases.

In conclusion, the Hippo-YAP signaling pathway represents a promising target for therapeutic intervention across a spectrum of skin disorders. As research continues to evolve, it is crucial to maintain a balanced and integrative perspective on the findings related to this pathway. Additionally, we can pave the way for novel treatment modalities that enhance our ability to manage and treat skin diseases, improving patient outcomes and quality of life. The future of dermatological therapeutics may very well hinge upon our continued exploration and understanding of the Hippo-YAP pathway and its multifaceted roles in skin health.
